# Is Malaria an Important Cause of Death among Adults?

**DOI:** 10.4269/ajtmh.20-0036

**Published:** 2020-04-20

**Authors:** Hellen Gelband, Isaac I. Bogoch, Peter S. Rodriguez, Michelle Ngai, Nazia Peer, Leah K. Watson, Prabhat Jha

**Affiliations:** 1 Centre for Global Health Research, Dalla Lana School of Public Health, St. Michael’s Hospital, University of Toronto, Toronto, Canada;; 2 Divisions of General Internal Medicine and Infectious Diseases, Toronto General Hospital, University Health Network, Toronto, Canada;; 3 Department of Medicine, University of Toronto, Toronto, Canada;; 4 SAR Laboratories, Sandra Rotman Centre for Global Health, Toronto General Hospital, University Health Network, Toronto, Canada;; 5 Global Strategy Lab, Faculty of Health and Osgoode Hall Law School, Dahdaleh Institute for Global Health Research, York University, Toronto, Canada

## Abstract

A long-held assumption has been that nearly all malaria deaths in high-transmission areas are of children younger than 5 years and pregnant women. Most global malaria mortality estimates incorporate this assumption in their calculations. In 2010, the Indian Million Death Study, which assigns cause of death by verbal autopsy (VA), challenged the reigning perception, producing a U-shaped mortality age curve, with rates rising after age 45 years in areas of India with substantial malaria transmission. Similar patterns are seen in Africa in the International Network for the Demographic Evaluation of Populations and Their Health (INDEPTH) network, also relying on VA. Whether these results are accurate or are misidentified deaths can be resolved by improving the evidence for assigning causes for adult acute infectious deaths in high malaria transmission areas. The options for doing so include improving the accuracy of VA and adding postmortem biological evidence, steps we believe should be initiated without delay.

## INTRODUCTION

Knowledge about malaria, from the molecular to the population level, is vast, much of it gained in the past half century. Lingering uncertainty about whether malaria is an important cause of death among adults living in endemic areas may, therefore, be surprising.

In this article, we review the available evidence supporting the possibility of a substantial burden of adult malaria mortality in India and Africa, discuss the reasons for skepticism about accepting malaria cause-of-death attribution in verbal autopsy (VA) studies, and suggest ways to narrow or eliminate the existing uncertainty. First, we discuss the general and malaria-specific state of cause-of-death data from malaria-endemic countries.

## MALARIA MORTALITY: REPORTS AND ESTIMATES

The countries in Africa and Asia with the greatest malaria burdens also have some of the poorest vital registration systems. Most sub-Saharan African countries report sporadic and fragmentary data, or none at all.^1^ The lack of reliable cause-of-death data has meant that, as Snow and others observed in 1999, “estimates of the health impact of diseases such as malaria have swung between semi-informed guesses and wild speculation.”^2^ They went on to say that “we know surprisingly little about malaria mortality among older children and nonpregnant adults under endemic conditions in Africa.” Little has changed since then, and the same is true outside of Africa.

The WHO publishes annual country-specific estimates of malaria mortality using one of three methods, depending on the quality and completeness of malaria data. The first method, used in most countries outside of Africa, including India, applies an average case fatality rate taken from published and some unpublished studies, to the number of estimated clinical cases of malaria.^3^ The second mortality estimation method, used for most African countries, is a model based on deaths in children younger than 5 years identified by VA, with parasite prevalence in the age-group as a covariate.^3^ This means that assumptions about age-specific malaria mortality are built into the estimates, making mortality data from these countries relatively uninformative about the shape of the curve. The third method of mortality reporting, using actual reported deaths, cannot be used in any highly endemic country.

It is worth noting that the incidence data on which mortality estimates are based are themselves estimates based on largely incomplete reporting of cases. While possibly the best available, WHO’s estimates embody substantial uncertainty, and trends in these estimates may be related equally to changes in data collection or interventions (e.g., introduction of rapid diagnostic tests) as to real changes in mortality.

In 2000, WHO estimated that malaria was causing between 1.1 and 2.7 deaths annually, about 1 million of them children younger than 5 years in sub-Saharan Africa.^4^ Similarly, 5 years later, the 2005 World Malaria Report attributed “more than one million” deaths in Africa to malaria, most of them in children younger than 5 years.^5^ Since then, the estimated number of childhood malaria deaths has declined dramatically, which reflects success of a decade of intense malaria control. By 2010, the global mortality estimate had fallen to about 600,000 deaths, about 75% of them estimated to be in under-fives, and by 2017, 435,000 deaths were estimated, about 60% of them in under-fives.^3^

Even these crude estimates leave open the possibility of substantial numbers of adult malaria deaths, but the clear emphasis is on deaths in childhood. In the early 2000s, in particular, investment in malaria control focused largely on reducing child deaths and illness, with prevention through the use of insecticide-treated nets and intermittent preventive therapy and treatment with effective antimalarial drugs. Pregnant women, who are also at risk of severe malaria, have also been included in intervention programs, particularly intermittent preventive treatment.^6^ More recently, interventions have been made available population wide, but global indicators are still focused almost entirely on children. The Malaria Indicator Survey, based on a household survey carried out in all endemic countries, collects data on what it considers “all of the internationally recognized malaria indicators,” all but one of which focus on children younger than 5 years and pregnant women. (The exception is indoor residual spraying of insecticides to kill mosquitoes, which would benefit everyone in the house.)^7^ The Malaria Atlas Project uses the *Plasmodium falciparum* parasite rate in 2- to 10-year-olds as their main metric for malaria burden.^8^

WHO’s *World Malaria Report 2019* makes no mention of age-specific malaria rates or of deaths among older people.^9^ Its main message is conveyed by a cover illustration of a pregnant woman with two children. The importance of protection during pregnancy and in childhood is undisputed, but it may not be the entire story.

## FINDINGS FROM VERBAL AUTOPSY STUDIES

### The Million Death Study.

**Figure 1.**
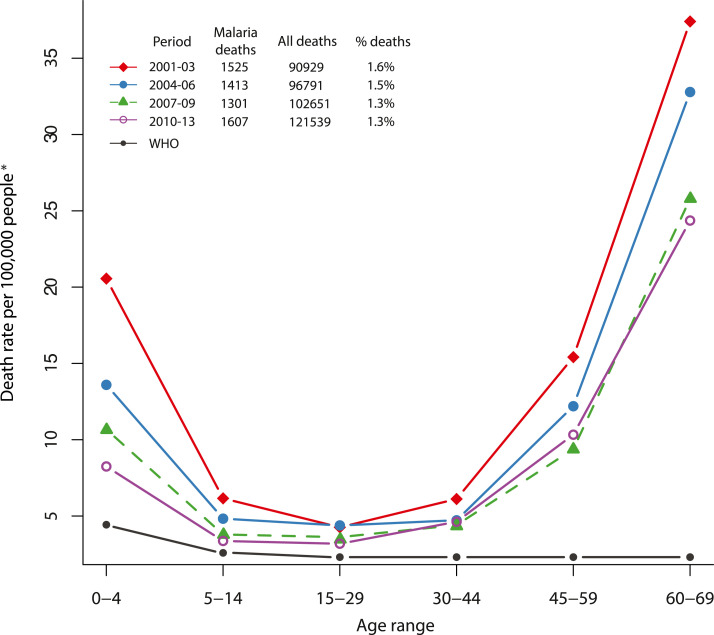
Malaria age-specific deaths rates in the Million Death Study (MDS), 2001–2013. Malaria mortality rates (per 100,000 person-years) in India from the MDS (four time periods) spanning 2001–2013 and as estimated indirectly by the WHO for 2006, by age-group. Million Death Study data for the four periods were standardized to United Nations deaths and population by Indian states for 2005, 2008, 2011, and 2015, respectively (Ref. 10 and unpublished MDS data). *Calculation of six age-specific death rates applied the above proportions of malaria to total deaths to the UN estimates of deaths (< 70 years, totals of 6.62 million, 6.44 million, 6.25 million, and 6.08 million for 2005, 2008, 2011, and 2015, respectively). The relevant population younger than age 70 years for these years were 1.11 billion, 1.16 billion, 1.21 billion, and 1.27 billion, respectively. This figure appears in color at www.ajtmh.org.

The 2010 Million Death Study (MDS) analysis for the years 2001–2003 was the first to suggest formally that adult malaria deaths may be more common than previously thought, producing a U-shaped age-specific mortality curve, with high death rates in infants and children and low rates in young adults through mid-life, rising again continually from about age 45 years (Figure 1).^10^ Of 75,342 deaths of people aged 70 years captured by nonmedical staff conducting structured VAs in 6,671 randomly selected areas of India, 2,681 (3.6%) were attributed to malaria after dual, independent physician assignment of deaths. About 60% of these malaria deaths were in “high malaria states.” This translates to a national estimate of 205,000 deaths per year, with nearly 60% (120,000) occurring after childhood, at ages 15–69 years. Both the total number of malaria deaths and the number among adults raised concern about relying on estimates based on selective data, or few data at all. At the time, the WHO estimated a total of 15,000 malaria deaths per year in India in 2006—an order of magnitude lower than the MDS estimate (Figure 1).^10,11^

Million Death Study deaths have now been captured and reported through 2013, and the U-shaped distribution clearly persists (Figure 1) (unpublished data from MDS). From 2004 to 2013, 7,527 (2.3%) deaths were attributed to malaria, representing about 150,000 deaths nationwide. Comparing high and low malaria states on the percentage of deaths from major causes for which “fever” was a symptom (Table 1), deaths from malaria stand out as the most discrepant—20% in high and 7% in low malaria states. A 5% higher distribution of acute respiratory infections in low versus high malaria states (21% versus 15%) could signal some malaria overdiagnosis but not enough to account for the entire difference. We believe this makes a compelling case for at least considering that malaria deaths among adults, particularly those dying at home, may be considerably more common than has been assumed.

**Table 1:** Fever-related cause-of-death distribution by high and low malaria regions, 2001–2013

Cause of death	Percentage of high malaria (*n*)	Percentage of low malaria (*n*)
Malaria	20.7 (2,660)	6.7 (3,467)
Acute respiratory infections	15.6 (1,998)	21.0 (10,827)
Diarrheal diseases	13.1 (1,686)	12.7 (6,566)
Tuberculosis	11.0 (1,407)	10.4 (5,336)
Fever of unknown origin	7.4 (951)	10.3 (5,310)
Hepatitis	5.1 (654)	3.0 (1,523)
Severe systemic infection	3.8 (493)	3.6 (1836)
Asthma and chronic obstructive pulmonary disease	1.9 (241)	4.4 (2,277)
Meningitis/encephalitis	1.5 (189)	3.5 (1,825)
Measles	1.0 (132)	2.4 (1,228)
Other	18.8 (2,412)	22.0 (11,331)
*TOTAL*	*100 (12,823)*	*100 (51,528)*

Million Death Study high malaria states are Odisha, Chhattisgarh, Jharkhand, Assam, and six smaller northeastern states. All other states comprise the low malaria group (Ref. 10 and unpublished MDS data).

We also compared the earlier MDS results (2001–2003) with information from other sources in India.^10^ The proportional distribution of malaria deaths in the MDS in states with substantial malaria transmission is similar to the geographic pattern of clinically confirmed malaria deaths reported by the national malaria control program. The same is true comparing malaria mortality from the MDS with *P. falciparum* district transmission rates from the National Vector Borne Disease Control Programme. The malaria mortality distribution was *not* correlated with national data for dengue, typhoid, or meningitis, which might be confused with malaria. The MDS deaths coded as “fever of unknown origin” also were not correlated with the independent malaria indices.

### The INDEPTH network.

The MDS findings represent India only. The INDEPTH network has generated population-based cause-of-death information since the 1990s at 49 health and demographic surveillance system field sites in Africa and Asia. Cause of death is assigned using the algorithms from InterVA-4,^12^ resulting in the largest available dataset of individually coded malaria age-specific mortality.

**Figure 2.**
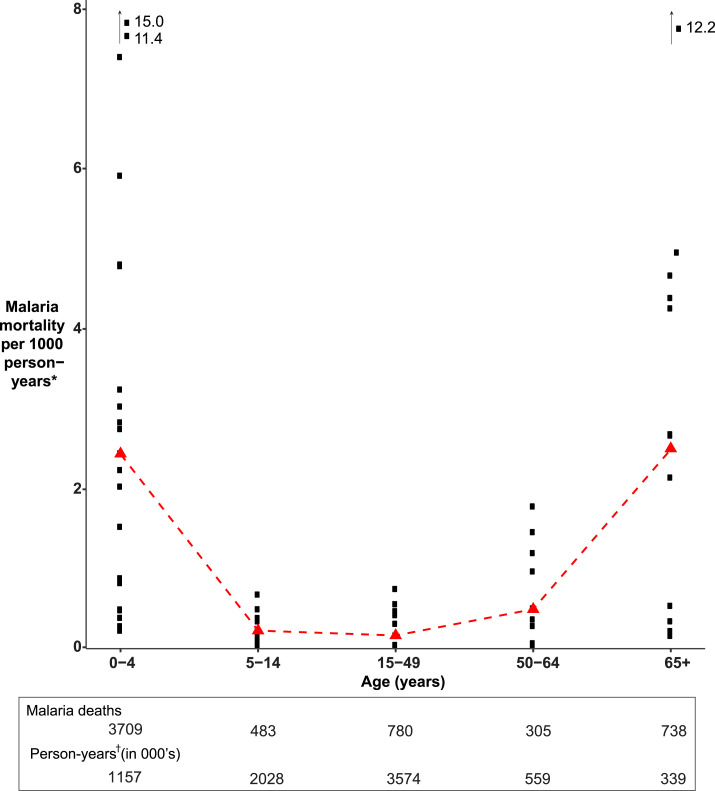
Malaria age-group–specific death rates in African INDEPTH sites, 2000–2012 data represent malaria deaths in almost all African INDEPTH sites from the combined periods of 2000–2005 and 2006–2012. The sites include Burkina Faso one (Nouna) and two (Ouagadougou); Cote d’Ivoire (Taabo); Ghana one (Navrongo) and two (Dodowa); Kenya one (Kilifi), two (Kisumu), and three (Nairobi); Senegal (Bandafassi); South Africa one (Agincourt) and two (South Africa Centre), combined because of small numbers of deaths; and the Gambia (Farafenni). The Ethiopia site was excluded because only 13 malaria deaths were reported for the entire period, and the Malawi site was excluded because it did not record child deaths. Malaria death rates were calculated by dividing the sum of weighted likelihoods by the sum of person-years across periods for each site and age-group (black squares). The red triangles represent the median value across all sites for each age group. Data come from INDEPTH (2014). Weighted likelihood and person-years are taken from the INDEPTH data set. The 0–4 years age-group excludes neonatal deaths (age 0–1 month). For more details, see 12. Data source: INDEPTH Data Repository (https://www.indepth-ishare.org/index.php/catalog/48). *Mortality rates were estimated using INDEPTH data and the same method as described by Streatfield et al.^12^ †Total person-years of exposure for all individuals under surveillance in the particular year, age-group, and gender. This figure appears in color at www.ajtmh.org.

The overall median age-group–specific malaria mortality rates for 2000–2012 for INDEPTH Africa sites appear to be similar to the U-shaped MDS pattern in India: high in children younger than 5 years and increasing in older adults, especially in the age-group of 65 years and older (Figure 2). Patterns at individual sites vary and not all are U-shaped, particularly the sites with lower malaria endemicity levels, which tend to be flatter (see Figure 2 in Streatfield et al., 2014^12^). The authors point out that the deaths attributed to malaria among older people by VA have “no associated biomedical evidence that these deaths are indeed directly due to malaria.” Indeed, nor do the childhood deaths, but the authors express some confidence in the results based on validity checks conducted. One check is a site-specific correlation of the mortality rates for adults (15 years and older) and children (younger than 15 years), yielding a strong correlation (*R*^2^ = 0.65). As observed by the INDEPTH authors:

If there were appreciable misclassification, the so-called “malaria” deaths in adults might be expected to occur at a rate largely independent of childhood malaria mortality, in the absence of any hypothesis as to other causes of acute adult febrile mortality that happened to correlate geographically with childhood malaria.^12^

### National census results in Africa.

An additional source of directly estimated age-specific malaria mortality comes from the *Sierra Leone 2015 Population and Housing Census Thematic Report on Mortality*, where malaria was the leading cause of death in 2015, accounting for 27% of deaths.^13^ The information was collected at the household level, but an impressive 93% of deaths had been documented and reported, leading to reasonable confidence in the reports. (The high level of reporting may be attributable to measures adopted during the Ebola epidemic, before the 2015 census.) The highest age-specific mortality rate is in the under-five age-group and the lowest in those aged 5––14 years. From there, the rate rises in the 15–49 years age-group and declines somewhat in the 50+ years age-group.

Similarly, a post-census mortality survey in conjunction with Mozambique’s census of 2007 conducted VAs on 10,080 deaths occurring from August 1, 2006 to July 31, 2007 with dual independent physician review of the causes of death.^14^ Malaria accounted for 29% of all deaths; about half of all deaths in children aged 28 days to 14 years and 14% of deaths in those aged older than 15 years. The Mozambique malaria curve was also U-shaped, albeit with much higher rates in younger children than in older adults.

### Limitations of VA studies.

The MDS and INDEPTH findings challenge current beliefs that malaria deaths in high-transmission areas are confined largely to children. However, the limitations of relying entirely on VA findings leave too much room for uncertainty. We can be sure that not all the deaths attributed to malaria by VA are actually malaria because some misclassification is inevitable. It is likely, as well, that some malaria deaths are misclassified as due to other causes. What we do not know is how large these errors are. We believe that the precision can be substantially improved, however, by two main routes: 1) improving the sensitivity and specificity of VAs for malaria deaths by better use of information on adult severe malaria symptomatology and contextual information and 2) analyses of postmortem biological samples.

## IMPROVED VERBAL AUTOPSIES FOR MALARIA AND OTHER LIKELY CAUSES OF ACUTE INFECTIOUS DEATHS IN ADULTS

Basic VAs are designed to cover all causes of death as accurately and efficiently as possible, and are, inevitably, more accurate for some causes than others,^15^ but they can be supplemented to study specific causes. By creating a targeted VA tool that harnesses clinical, epidemiologic, seasonal, and biologic data, we should be able to substantially narrow the uncertainty of the causes of adult death from acute febrile illnesses, including malaria. A successful example is a small supplemental VA tool, the “AIDS Treatment Module” (ATM), which was devised as an add-on to the WHO VA to better understand causes of death related to HIV infection in southern African settings.^16,17^ The ATM signals why people die from HIV and can identify factors that contributed to death, for example, lack of access to or poor adherence to HIV medications.

A VA designed to distinguish malaria from other acute febrile illnesses with overlapping signs and symptoms poses several challenges. The differential diagnosis is broad and includes diseases such as influenza, arboviruses, rickettsiae, infectious causes of meningitis, and bacterial sepsis syndromes (including infection with invasive salmonelloses and leptospirosis).^18,19^ The probability of a particular pathogen causing illness varies geographically, is dynamic, and may change based on the setting and season. These variations are recognized and can be incorporated into a more precise VA for adult causes of death from acute febrile illness.

Ideally, a malaria-specific VA would be based on observations of home deaths with no medical attention, but, for obvious reasons, such information is almost nonexistent. Some severe malaria patients do reach hospitals in low-resource areas, some dying and some surviving. In addition to useful information in the clinical records of such cases, the clinicians and nurses who have cared for these patients can provide valuable observations of signs and symptoms to inform the VA instrument. Other data sources exist as well. Some published literature describes hospital-based adult malaria deaths in Africa and Asia. Equally valuable (and more numerous) are clinical descriptions representing both survival and death from malaria in intensive care units (ICUs) in high-income countries, of both nonimmune returning tourists and semi-immune African and Asian nationals arriving in the new country and quickly falling gravely ill.^20,21^ These are individuals who may well survive but would almost certainly die without the kind of support provided in an ICU, such as renal dialysis, mechanical ventilation, or vasopressors.

Based on review of both African and Asian hospitals, and any ICU reports, it is apparent that adults die (or require ICU care) from malaria infection because of complications of acute respiratory distress syndrome (ARDS), cerebral malaria, metabolic acidosis, renal failure, and sepsis syndromes—and typically multiple processes.^22–26^ Malaria infection during pregnancy predisposes individuals to succumb from these same disease processes.^27^

A dedicated VA tool could be designed to detect clinical signs of these end-stage disease processes in adults. For example, questions can target ARDS (e.g., respiratory failure in the absence of sputum production), metabolic acidosis (e.g., tachypnea in the absence of cough), cerebral malaria (e.g., seizures, altered mental state, and headache), and acute renal failure (e.g., anuria, anasarca, and encephalopathy), or combinations of these processes. The precision of a dedicated malaria VA tool could be strengthened by incorporating supplementary epidemiologic and biologic data. For example, both malaria and bacterial infections may cause meningitis syndromes; however, in West Africa, bacterial meningitis is much more common between February and May and very infrequent during other months, whereas malaria cases are far more common between August and November.^28,29^ Even in regions with year-round malaria transmission such as Jharkhand, India, there is still a seasonal rise in malaria incidence from May through September.^30^

## POSTMORTEM BIOLOGICAL TESTING

An improved VA may reduce the uncertainty around the cause of a death due to an acute febrile illness, but it cannot be definitive. Assuming that it is possible to overcome the formidable logistical hurdles of obtaining viable postmortem samples from people who have died outside of medical facilities, several types of blood-based assays could contribute to a more confident determination. These include point-of-care tests developed for diagnosis of live patients and the expanding array of host severity markers identified in the fast-moving field of biomarker research. None of these markers has been studied for postmortem use, and there is little to no information on their postmortem characteristics. Our approach is to try to validate what we expect will be a small set of host biomarkers that could 1) confirm with high specificity that the death was due to a severe infection and 2) differentiate a malaria death from death due to other likely infectious causes with substantially less uncertainty than possible with a positive (preferably quantitative) postmortem malaria test and VA alone. This will be a novel use of biomarkers, including those indicating a generic response to severe infection and others closely associated with malaria (and eventually, other likely causes).

Biomarkers that signal severe, possibly life-threatening, episodes in response to many or most pathogens have been reliably identified in the search for clinically useful indicators to quickly triage patients who need supportive, life-saving treatment even without identifying a specific cause. One of the shared pathways that contribute to the pathogenesis of severe and fatal infections is endothelial and immune activation, which precedes the loss of endothelial integrity, microvascular leakage, multiorgan dysfunction, and ultimately death.^31^ Markers of these pathways (angiopoietin/receptor tyrosine kinase Tie2 and soluble triggering receptor expressed on myeloid cells-1) have been shown to be independent, quantitative markers of disease severity and prognosis in malaria, sepsis, and other infections.^31,32^

Identification of malaria-specific markers is more recent and ongoing. In a study of 390 Gambian children, haptoglobin and lipocalin-2 were able to discriminate with high accuracy between acute respiratory infection and severe malaria with respiratory distress (area under receiver operating characteristics (AUROC) = 0.99). The utility of the two markers was further validated in a second cohort of Kenyan children (AUROC = 0.82).^33^ Haptoglobin was more recently included in a three-marker panel that was able to discriminate between bacterial infection and malaria with 96% sensitivity and 86% specificity.^34^ Further validation studies are under way. Both generic and disease-specific markers require further validation, including determining their fate postmortem. Preliminary work can be conducted in mice, but pilot studies under field conditions will also be necessary.

We have also considered the use of minimally invasive tissue sampling beyond blood samples, which has been adopted by a growing list of studies in low-resource settings.^35^ Our initial strategy will be to focus on blood alone in pilot testing because of the considerably greater logistical challenges of collecting and analyzing solid tissue samples.

## DISCUSSION

Understanding the causes of death in populations is a cornerstone—even the bedrock—of public health and medicine. Knowing who dies of what has led to much of the progress of the past two centuries in preventing premature mortality from a range of causes. We believe there is considerable value in understanding the age-specific patterns of death from malaria, as the world strives to eliminate the disease.

Unfortunately, the countries with the greatest malaria burden tend of have poorly functioning vital registrations systems, leading to the types of mortality estimates described earlier. Sample registration systems, exemplified by the MDS, and INDEPTH’s site-specific surveillance, both of which rely on VA for cause-of-death determination, provide an intermediate level of data, but also raise questions. The issue of possibly higher-than-expected adult malaria mortality is an example. And if these deaths are largely misidentified as malaria, what are the true causes? Whatever is true, it could and should affect how severe acute infections in adults are treated (or preferably, prevented). We have not pinpointed age-specific service provision gaps for this cohort, but contend that, at least in many places, malaria may not be routinely considered in the differential diagnosis in adults presenting with fever. We agree with Streatfield and others,^12^ who, based on their INDEPTH analysis, suggest that “[b]ecause malaria surveillance among older people has generally not been given high priority, there appears to be a need for further population-based research” on the question.

Skepticism of VA results alone is healthy. The arguments against believing that malaria is causing substantial deaths are well reasoned. People know when they live in an area of high malaria transmission. When interviewed about an acute infectious death, they may be inclined to have a heightened memory of what they consider malaria-like symptoms, which is conveyed to the interviewer. Although interviewers should not influence the interviewees, it would not be unheard of for this to happen. Finally, if physicians are coding cause of death, they may have an unconscious bias toward a malaria determination if they know that the deceased comes from a high-transmission area. For computer-coded VAs, a bias could even be programmed in.

Given the limits of VA, we have searched for reasons to discount or diminish the unexpectedly high malaria mortality rates among adults in the MDS (although we have not done the same for INDEPTH). In India, we found no deficits of other likely causes, including dengue, meningitis, pneumonia, tuberculosis, or HIV, but we have found a lower percentage coded as fever of unknown origin in high-transmission areas. We have found that the deaths correlate with other malaria indices (but this could be ascribed to the bias aforementioned). We are not attempting to explain the mechanisms (presumably immunologic) underlying the patterns we describe, but note that arguments against the observed excess being due to malaria are based in large part on an assumption that acquired immunity to malaria is unchanging even at older ages.^36^ While involving different aspects of the immune response than malaria immunity, immunity to other common infections, such as influenza and pneumonia, does wane with age.^37,38^

With the low level of vital event reporting from most countries with significant malaria burdens, the level of uncertainty about causes of death is not surprising, and similar questions could be asked equally appropriately about other causes. We believe that the aspiration toward malaria elimination and the impressive set of effective interventions available to prevent and treat malaria elevate its importance at this moment.^39^ We also believe that an evidence-based add-on VA module focused on the symptomatology of severe malaria in adults can be developed that will much more clearly distinguish malaria from other infectious causes of death, but that biological confirmation is also needed to further reduce the uncertainty. For practical reasons and encouraged by completed and ongoing research, we believe that it should be possible to identify a set of blood-borne severity and parasite-related biomarkers that can help rule in malaria and rule out important differential diagnoses. The approach outlined—which we are actively pursuing—is a practical and feasible alternative to the current uncertainty surrounding the question of adult malaria deaths.
